# Fluid Homeostasis May Predict the Prognosis of Non-infectious Fever After Total Knee Arthroplasty Within 7-Day: A Retrospective Cohort Study

**DOI:** 10.3389/fsurg.2021.690803

**Published:** 2021-09-17

**Authors:** Nafei Xu, Taotao Xu, Xiaoxue Tan, Lujie Xu, Menghua Ye, Yue Pan, Peijian Tong, Xueqin Hu, Min Xu

**Affiliations:** ^1^School of Nursing, Zhejiang Chinese Medical University, Hangzhou, China; ^2^Department of Health and Tourism, Hangzhou Wanxiang Polytechnic, Hangzhou, China; ^3^Department of Orthopedic Surgery, The First Affiliated Hospital of Zhejiang Chinese Medical University, Hangzhou, China; ^4^Department of Hematology, The First Affiliated Hospital of Zhejiang Chinese Medical University, Hangzhou, China; ^5^Department of Psychiatry, Faculty of Medicine & Dentistry, University of Alberta, Edmonton, AB, Canada; ^6^Institute of Orthopadics and Traumatology, The First Affiliated Hospital of Zhejiang Chinese Medical University, Hangzhou, China; ^7^Department of Administration, The First Affiliated Hospital of Zhejiang Chinese Medical University, Hangzhou, China

**Keywords:** total knee arthroplasty, risk factors, nomogram, fluid homeostasis, non-infectious fever

## Abstract

**Background:** In the perioperative management of Total Knee Arthroplasty (TKA), postoperative fever has always been a concern. Current research focuses on infectious fever, and there is no relevant research on the occurrence of non-infectious fever (NIF) and its risk factors. Hence, the aim of this study was to clarify the risk factors for NIF after TKA, and construct an easy-to-use nomogram.

**Methods:** A retrospective cohort study was conducted. Consecutive patients undergoing primary unilateral TKA were divided into the non-infectious fever group and the control group. Clinicopathological characters were collected from electronic medical records. Univariate Logistic regression was used to analyze the related independent risk factors. The optimal threshold for each selected factor and combined index was determined when the Youden index achieved the highest value. And the predictive nomogram was developed by these independent factors.

**Results:** Ultimately, 146 patients were included in this study. Of them, 57 (39.04%) patients experienced NIF. Results of the univariable logistic regression analysis indicated that intraoperative blood loss (OR, 1.002; 95% CI, 1.000–1.0004), postoperative drainage fluid volume (OR, 1.003; 95% CI, 1.001–1.006) and frequency of blood transfusion (*n* = 1; OR, 0.227; 95% CI, 0.068–0.757) were independent risk factors of NIF occurrence. The predictive nomogram that incorporated the above independent risk factors was developed, and it yielded an areas under the curves (AUC) of 0.731 (95% CI: 0.651–0.801; *P* < 0.0001) with 54.39% sensitivity and 82.02% specificity.

**Conclusions:** Non-infectious fever after TKA prolongs the time of antibiotic use and hospital stay. Our results demonstrated that the nomogram may facilitate to predict the individualized risk of NIF occurrence within 7-day by intraoperative blood loss, postoperative drainage fluid volume and frequency of blood transfusion.

## Introduction

Total Knee Arthroplasty (TKA) can improve joint mobility, correct deformities and restore joint function, and is recognized as the best treatment option for end-stage osteoarthritis (OA) (1–3). With the improvement of the quality of life for OA patients, the demand for knee replacement has greatly increased (4). In the perioperative management of this surgery, postoperative fever has always been a concern (5, 6). Infectious fever (IF) has been paid much more attention by scholars whereas non-infectious fever (NIF) has much less (7). In fact, non-infectious fever, majority of the fever after arthroplasty, is common in clinical practice (8). Non-infectious fever usually refers to the negative bacterial culture of blood, urine, sputum and joint cavity puncture fluid accompanied by fever symptoms (9). Limited studies have found that non-infectious factors such as tissue damage, drug and blood transfusion reactions, anemia, and deep vein thrombosis can all stimulate the body's release of inflammatory mediators and increase body temperature, but the primary and secondary relations of various factors is still unclear (10). Moreover, there are currently no reliable markers to predict the prognosis of NIF.

Clinically, patients with postoperative fever may prompt expensive invasive tests to determine whether it is an infection, but studies have shown that 76–98% of patients have negative test results (11). It can be seen that non-infectious fever is the main body of fever after TKA, and current research focuses on infectious fever, and there is no relevant research on the occurrence of non-infectious fever and its risk factors. By analyzing the risk factors of non-infectious fever after TKA, the perioperative management plan for elective surgery can be optimized, the occurrence of postoperative fever can be reduced, and the patient's prognosis can be improved.

Connecting with the practical situation, this paper probes into the easily monitored risk factors, which could affect fluid balance and blood volume, like intraoperative blood loss, blood transfusion and postoperative drainage fluid volume. These factors correlated significantly with fluid homeostasis of the organism, a key factor in maintaining thermoregulatory capacity (12, 13).

Therefore, the clinical data of patients undergoing primary unilateral knee replacement in our hospital were collected retrospectively in this study, aiming to clarify the risk factors for non-infectious fever of primary unilateral TKA, and constructed an easy-to-use nomogram for the risk of occurrence.

## Methods

### Participants

From July 2018 to December 2019, 183 patients undergoing knee replacement were admitted to the Department of Orthopadics and Traumatology of the First Affiliated Hospital of Zhejiang Chinese Medical University.

*Inclusion criteria* included: (1) Diagnosed as knee osteoarthritis; (2) Using artificial knee joint replacement or total knee replacement; (3) First unilateral surgery; (4) Postoperative chest radiograph, hematuria, articular cavity puncture and other examinations showed no obvious signs of infection. *Exclusion criteria* included: (1) Patients undergoing unicondylar replacement surgery; (2) Severe perioperative complications, such as myocardial infarction, stroke, diffuse intravascular coagulation disorder, shock or pulmonary embolism, etc.; (3) Incomplete case data records.

### Data Collection

#### Baseline Data and Drug Use Before TKA

Gender, age, diagnosis, past medical history (hypertension, diabetes, hyperlipidemia, cerebrovascular disease, etc.), preoperative chest radiograph, blood routine, urine routine and medication use (antibiotics, non-steroidal anti- inflammatory analgesics).

#### Operation Record

Surgeon, operation time, operation duration, prosthesis company, intraoperative blood loss, and blood transfusion.

#### Stay of Hospital, Postoperative Complications, and Related Parameters

Total length of hospital stay, postoperative complications (arrhythmia, myocardial infarction, stroke, diffuse intravascular coagulopathy, shock or pulmonary embolism, etc.), postoperative drainage fluid volume, postoperative body temperature and duration of antibiotic use.

From the day after the operation to the 7th day, the nurse used an infrared tympanic thermometer to collect and record the patient's body temperature. The temperature is measured at least 4 times a day (6:00, 10:00, 14:00, 18:00). In addition, this study defines non-infectious fever as: the body temperature measured at any point within 7-days after surgery is equal to or higher than 38.0°C, and negative chest x-ray, urine culture, blood culture, or other interventions included joint aspiration, Doppler ultrasound, or chest computed tomography (14).

The study was approved by the Ethics Committee of the First Affiliated Hospital of Zhejiang Chinese Medical University (Ethical approval ID: 2018-KL-018-01).

### Statistics

Data were analyzed using Statistical Package for Social Sciences (SPSS Windows version. 21.0; SPSS Inc., Chicago, IL., USA) R statistical software (version 3.3.3, https://www.r-project.org). Combined with the normal test chart, Kolmogorov-Smirnov (K-S) results are used to determine whether the measurement data obey a normal distribution. Normally distributed measurement data is represented by the Mean ± Standard Deviation (M ± SD), and skewed distribution measurement data is represented by the Mean (25 percentile, 75 percentile), that is, M (P25, P75). For comparison between groups, independent-sample *t*-test and Mann-Whitney U test were used for measurement data according to whether they obey normal distribution, and chi-square test was used for count data. For the variables with statistical significance, the two-class single-factor Logistic regression was used to analyze the related independent risk factors. Receiver operating characteristic (ROC) curves, including the area under the areas under the curves (AUC) and its 95% confidence interval (95% CI), were analyzed to evaluate the performance of prognostic prediction. The optimal threshold for each selected factor and combined index was determined when the Youden index achieved the highest value (15). Based on the differences, the optimal model was selected, and then parameter estimates of this model were then used for constructing a nomogram using the “rms” package (16). Statistical significance was set at α = 0.05.

## Results

**Figure 1:**
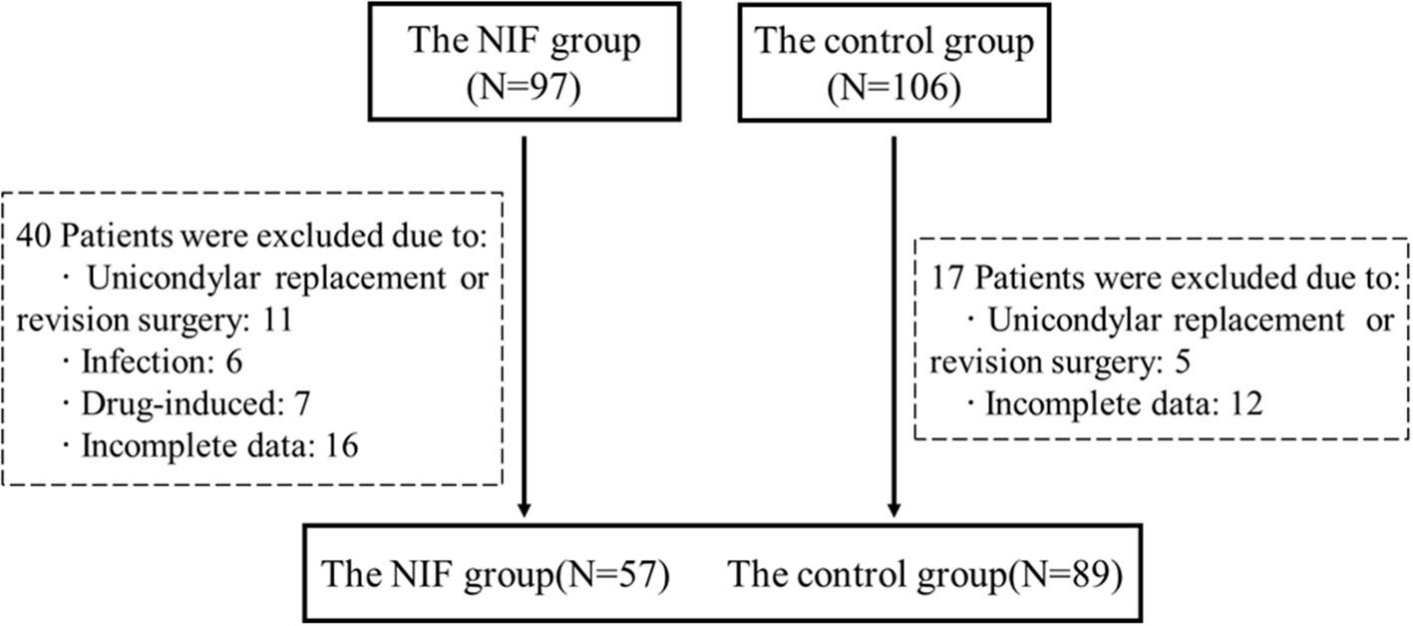
This is a flow diagram illustrating patient inclusion. NIF, non-infectious fever.

Verified the 203 selected participants, 16 patients underwent knee unicondylar replacement or revision surgery, 13 patients had postoperative fever caused by specific drugs for osteoporosis or infection, and 28 patients had incomplete data. Finally, 146 patients were included in this study (Figure 1).

### Clinical Characteristics of Study Subjects

Demographics are available in Table 1 and are summarized here. The mean age of participants was 66.71 ± 8.89 years (range, 44–81 years); the majority of participants were female (78.08%, 114/146) and male (21.91%, 32/146); 57 (39.04%) patients experienced NIF. According to the presence or absence of the primary endpoint, the patients were categorized into the non-infectious fever group (57 patients) and the control group (89 patients). There was no difference in the demographic characteristics between the two groups for age, gender or comorbidities.

**Table 1:** Comparison of relative factors between the non-infectious fever group and the control group after total knee arthroplasty.

**Characteristic**	**The non-infectious fever group (*N* = 57)**	**The control group (*N* = 89)**	**Statistics**	***P-*value**
Gender (male/female)	9/48	23/66	2.052	0.152
Age	66.36 ± 8.64	66.93 ± 9.09	−0.373	0.710
Hypertension	30	44	0.142	0.707
Diabetes	9	16	0.117	0.732
Operation duration (min)	193.95 ± 45.264	196.01 ± 41.678	−0.282	0.778
Intraoperative blood loss (ml)	363.33 (200, 500)	270.78 (150, 400)	−2.780	*0.005* ^*^
Postoperative drainage fluid volume (ml)	259.73 (100, 280)	151.30 (80, 170)	−4.822	*0.001* ^*^
Blood transfusion	32 (56.14%)	37 (30.33%)	2.958	0.085
Frequency of blood transfusion (≥2)	12 (21.05%)	5 (5.61%)	8.046	*0.005* ^*^
Duration of antibiotic use (day)	8.87 (4, 9)	5.52 (4, 7.5)	−2.429	*0.015* ^*^
Total length of hospital stay (day)	26.12 ± 9.517	23.34 ± 6.635	2.075	*0.04* ^*^

* *Significant at α level P <0.05*.

Intraoperative blood loss, postoperative drainage fluid volume, frequency of blood transfusion, duration of antibiotic use and total length of hospital in the control were significantly different from those in the NIF group, however, blood transfusion and operation duration were similar.

### Risk Factors of NIF Occurrence

Then, we performed further univariable logistic analysis using the factors with a *P* < 0.05 in the comparison between groups. Results of the univariable logistic regression analysis indicated that intraoperative blood loss (OR, 1.002; 95% CI, 1.000–1.0004), postoperative drainage fluid volume (OR, 1.003; 95% CI, 1.001–1.006) and frequency of blood transfusion (*n* = 1; OR, 0.227; 95% CI, 0.068–0.757) were independent risk factors of NIF occurrence, and display the results with a forest map (Table 2).

**Table 2:** Univariate logistic regression analysis of risk factors.

**Covariates**	***P*-value**	**OR**	**95%CI**		**OR(95%CI) plot**
			**Lower**	**Upper**	
Intercept	0.336	0.503			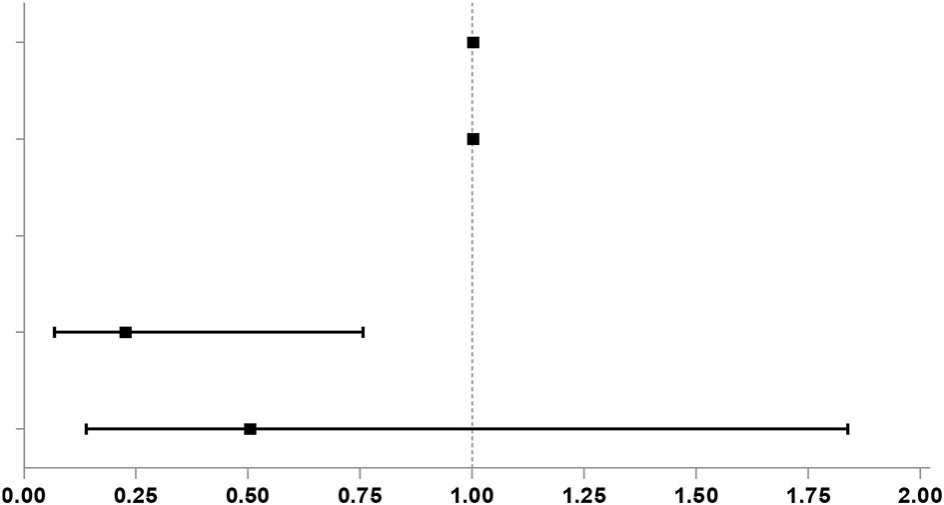
Intraoperative blood loss	0.030^*^	1.002	1.000	1.004	
Postoperative drainage volume	0.014^*^	1.003	1.001	1.006	
Frequency of blood transfusion	0.021^*^				
i 1	0.016^*^	0.227	0.068	0.757	
≥2	0.300	0.505	0.139	1.838	

*95% CI, 95% confidence interval*.

* *P <0.05*.

### Predictive Performance of the Intraoperative Blood Loss, Postoperative Drainage Fluid Volume, Frequency of Blood Transfusion and Combined Index

**Figure 2:**
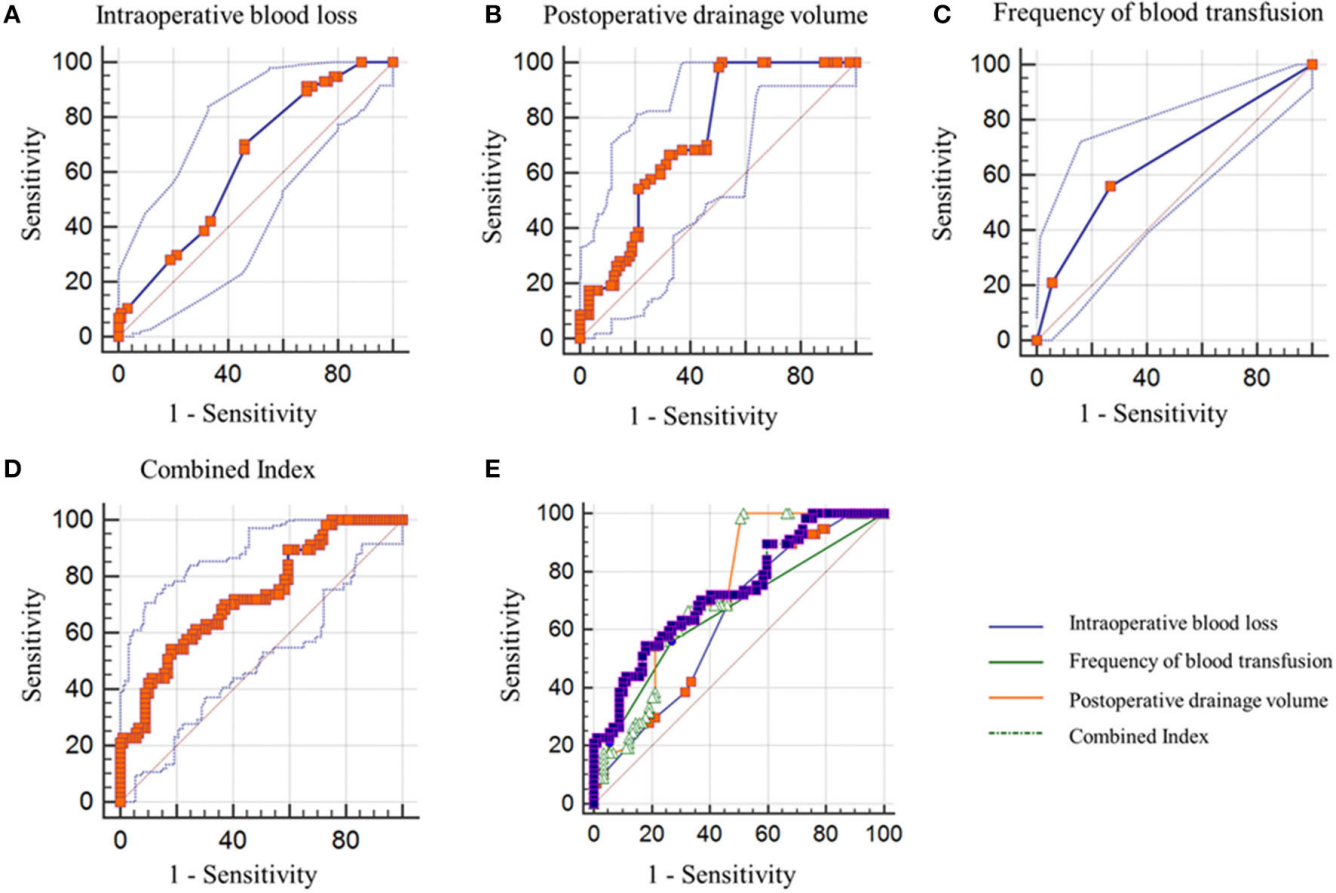
ROC curve analysis using the intraoperative blood loss **(A)**, postoperative drainage fluid volume **(B)**, frequency of blood transfusion **(C)**, and Combined Index **(D)** for predicting the 7-day endpoint. Combined Index was chosen as the optimal model **(E)**. ROC, receiver operating characteristic.

ROC curve analysis was used to analyze the predictive performance of the intraoperative blood loss, postoperative drainage fluid volume, frequency of blood transfusion and Combined Index (Figure 2). The optimal cut-offs and corresponding sensitivity and specificity are listed in Table 3. As expected, Combined Index yielded an AUC of 0.731 (95% CI: 0.651–0.801; *P* < 0.0001) with 54.39% sensitivity and 82.02% specificity, suggesting that the prognostic model had good sensitivity and specificity in predicting patients 7-day endpoint.

**Table 3:** Predictive performance of the intraoperative blood loss, postoperative drainage fluid volume, frequency of blood transfusion and Combined Index.

**Variables**	**Cut-off**	**AUC (95% CI)**	**Sensitivity**	**Specificity**	***P-*value**
Intraoperative blood loss	0.2411	0.635 (0.551–0.713)	70.18%	53.93%	*0.0028* ^*^
Postoperative drainage fluid volume	0.4831	0.736 (0.657–0.806)	100%	48.31%	* <0.0001* ^*^
Frequency of blood transfusion	0.2917	0.658 (0.576–0.735)	56.14%	73.03%	*0.0001* ^*^
Combined Index	0.3641	0.731 (0.651–0.801)	54.39%	82.02%	* <0.0001* ^*^

*AUC, areas under the curves; 95% CI, 95% confidence interval*.

* *P <0.05*.

### Nomogram Construction

**Figure 3:**
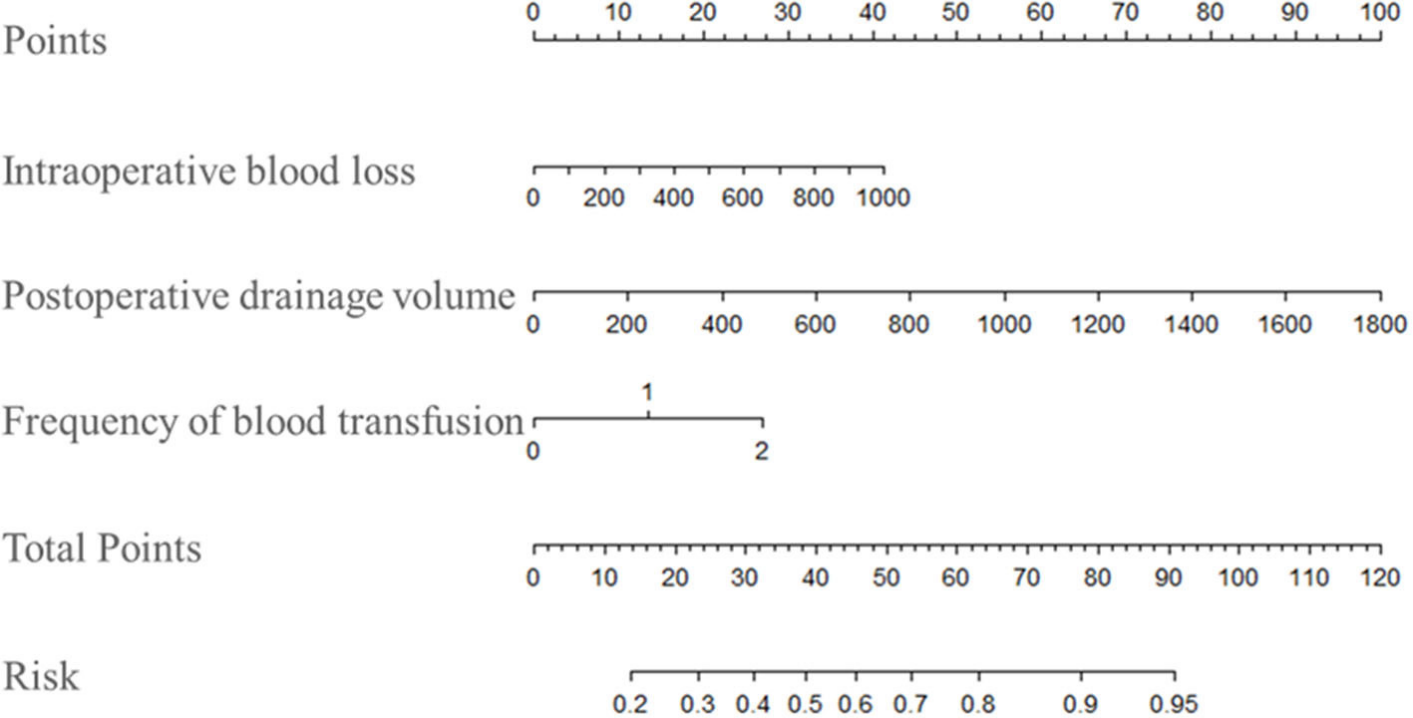
Nomogram plot of NIF probabilities within 7-day was demonstrated. NIF, non-infectious fever.

The predictive nomogram that incorporated the above independent risk factors was developed and is depicted in Figure 3. For the Clinical medical and nursing staff, the plot was available to locate a patient's intraoperative blood loss, postoperative drainage fluid volume and frequency of blood transfusion in each axis; to draw a line straight upward to the point axis and sum up the total points; and then to draw a line straight downward to determine the patient's risk for non-infectious fever of primary unilateral TKA. For example, a patient with a 600 ml intraoperative blood loss (25 points), 800 ml postoperative drainage fluid volume (45 points) and one time blood transfusion (15 points) underwent primary unilateral TKA, his total occurrence points were 75, with an approximated 7-day non-infectious fever occurrence probability of 88%. The ROC curve depicted in Figure 2D shows that derived nomogram performed well when compared to the actual results (AUC = 0.731).

## Discussion

To the best of our knowledge, this is the first study to explore the occurrent risk factors and construct an easy-to-use nomogram for NIF. This study retrospectively analyzed 146 patients after TKA, 57 patients developed non-infectious fever after surgery, the incidence was 39.04%, which was in line with the previous literature reports of 40%-50% (10). And the study clarified the harm of fever to patients, including prolonging the hospital stay of patients after TKA and the duration of antibiotic use. In addition, clinical observation found that non-infectious fever can cause panic in patients, affect doctors' clinical judgment or other interventions may have been performed.

Compared with non-infectious fever, transient postoperative hyperthermia has self-healing tendency, will not repeat, and is easy to be controlled by preventive antibiotics, However, non-infective fever is characterized by persistent POF and negative CXR, UC, and BC.

In the present study 11 related factors were included, results of the univariable logistic regression analysis indicated that intraoperative blood loss, postoperative drainage fluid volume and frequency of blood transfusion were independent risk factors of NIF occurrence. This result is similar to the results of Lu et al. (17), Kennedy et al. (18), Ishii et al. (19), and the incidence of fever after TKA is relatively high in cases of body fluid imbalance. However, Pan et al. found that reducing allogeneic blood transfusion may help prevent fever complications, especially in large orthopedic surgery (20). In addition, the amount of fluid drained after surgery is usually related to local vascular damage, wound exudation and metal stimulation. Andres et al. found that the bleeding and exudation of the surgically injured area will stimulate local inflammation, promote tumor necrosis factor, interleukin and other cytokines in the body, promote the production of endogenous heat sources, and cause the patient's body temperature to rise (21).

In addition, the entire research cycle is controlled within 2 years, and operation duration between the two groups were similar, which reduces the variable differences between the two groups to a certain extent and improves the reliability of the research results. However, there are several drawbacks to this study. First, as a retrospective design, our study has a certain selection bias; Second, as a single-center study, the number of included cases is small; Third, the included factors are still not comprehensive enough, and the relevant parameters of medical records, such as various inspection and laboratory results, are not fully included. Therefore, its clinical value requires further verification.

## Conclusion

To sum up, non-infectious fever after TKA prolongs the time of antibiotic use and hospital stay, which seriously affects the medical cost after surgery. We developed a nomogram to predict the individualized risk of NIF occurrence by intraoperative blood loss, postoperative drainage fluid volume and frequency of blood transfusion. This nomogram was well-fitted. Our well-fitted nomogram suggests that physicians should pay attention to fluid balance and weigh blood transfusion decisions, which is of great significance to reduce non-infectious fever after TKA.

## Data Availability Statement

The data used to support the findings of this study are available from the corresponding author upon request.

## Ethics Statement

The study was approved by the Ethics Committee of the First Affiliated Hospital of Zhejiang Chinese Medical University. Written informed consent from patients/participants was not required for this study in line with national and institutional requirements.

## Author Contributions

MX and XH: conception and design. MX and PT: administrative support. TX and PT: provision of study materials or patients. NX, MY, and YP: collection and assembly of data. NX, XT, and LX: data analysis and interpretation. All authors manuscript writing and final approval of manuscript.

## Funding

This work was supported by the National Natural Science Foundation of China (Grant Nos. 81603718, 81904223); Research Fund of Zhejiang Chinese Medical University (No. 2019ZR01); Medical Health Science and Technology Program of Zhejiang Province (No. 2020KY659); Youth Research and Innovation Fund of Zhejiang Chinese Medical University (No. KC201933); Bethune Charitable Foundation (No. G-X-2020-1107-17); Talent Cultivation Project of Zhejiang Association for Science and Technology (No. CTZB-2020080127).

## Conflict of Interest

The authors declare that the research was conducted in the absence of any commercial or financial relationships that could be construed as a potential conflict of interest.

## Publisher's Note

All claims expressed in this article are solely those of the authors and do not necessarily represent those of their affiliated organizations, or those of the publisher, the editors and the reviewers. Any product that may be evaluated in this article, or claim that may be made by its manufacturer, is not guaranteed or endorsed by the publisher.

## References

[1] Meiyappan KP Cote MP Bozic KJ Halawi MJ. Adherence to the American Academy of Orthopaedic Surgeons clinical practice guidelines for nonoperative management of knee osteoarthritis. J Arthroplasty. (2020) 35:347–52. 10.1016/j.arth.2019.08.051 31563393

[2] Jevsevar DS Brown GA Jones DL Matzkin EG Manner PA Mooar P. The American Academy of Orthopaedic Surgeons evidence-based guideline on: treatment of osteoarthritis of the knee, 2nd edition. J Bone Joint Surg Am. (2013) 95:1885–6. 10.2106/00004623-201310160-00010 24288804

[3] Felson DT. Clinical practice. Osteoarthritis of the knee. N Engl J Med. (2006) 354:841–8. 10.1056/NEJMcp051726 16495396

[4] Glyn-Jones S Palmer AJ Agricola R Price AJ Vincent TL Weinans H. Osteoarthritis. Lancet. (2015) 386:376–87. 10.1016/S0140-6736(14)60802-3 25748615

[5] Karam JA Zmistowski B Restrepo C Hozack WJ Parvizi J. Fewer postoperative fevers: an unexpected benefit of multimodal pain management? Clin Orthop Relat Res. (2014) 472:1489–95. 10.1007/s11999-014-3555-4 24615425 PMC3971226

[6] Ascione T Balato G Boccia G De Caro F. Predictive value of fever following arthroplasty in diagnosing an early infection. Infez Med. (2017) 25:3–7. 28353448

[7] Ward DT Hansen EN Takemoto SK Bozic KJ. Cost and effectiveness of postoperative fever diagnostic evaluation in total joint arthroplasty patients. J Arthroplasty. (2010) 25:43–8. 10.1016/j.arth.2010.03.016 20452174

[8] Czaplicki AP Borger JE Politi JR Chambers BT Taylor BC. Evaluation of postoperative fever and leukocytosis in patients after total hip and knee arthroplasty. J Arthroplasty. (2011) 26:1387–9. 10.1016/j.arth.2010.12.024 21353453

[9] Dorhout Mees SM Luitse MJ van den Bergh WM Rinkel GJ. Fever after aneurysmal subarachnoid hemorrhage: relation with extent of hydrocephalus and amount of extravasated blood. Stroke. (2008) 39:2141–3. 10.1161/STROKEAHA.107.509851 18436870

[10] Athanassious C Samad A Avery A Cohen J Chalnick D. Evaluation of fever in the immediate postoperative period in patients who underwent total joint arthroplasty. J Arthroplasty. (2011) 26:1404–8. 10.1016/j.arth.2011.02.019 21477972

[11] Ghosh S Charity RM Haidar SG Singh BK. Pyrexia following total knee replacement. Knee. (2006) 13:324–7. 10.1016/j.knee.2006.05.001 16806940

[12] Weinberg L Li M Churilov L Armellini A Gibney M Hewitt T. Associations of fluid amount, type, and balance and acute kidney injury in patients undergoing major surgery. Anaesth Intensive Care. (2018) 46:79–87. 10.1177/0310057X1804600112 29361260

[13] Shetty V Kasture S. Is immediate postoperative fever related to drop in haemoglobin? A comparative study in simultaneous bilateral total knee arthroplasty patients. Eur J Orthop Surg Traumatol. (2013) 23:345–7. 10.1007/s00590-012-0969-0 23412282

[14] Yao YT Li LH Lei Q Chen L Wang WP Chen WP. Noninfectious fever following aortic surgery: incidence, risk factors, and outcomes. Chin Med Sci J. (2009) 24:213–9. 10.1016/S1001-9294(10)60004-1 20120767

[15] Zhang W Wang R Ma W Wu Y Maskey N Guo Y. Systemic immune-inflammation index predicts prognosis of bladder cancer patients after radical cystectomy. Ann Transl Med. (2019) 7:431. 10.21037/atm.2019.09.02 31700867 PMC6803204

[16] Hu J Li C Guo X Zhang H Li H Qiu D. Development and validation of a predictive nomogram for the risk of recurrence in patients with cystitis glandularis. Ann Transl Med. (2020) 8:352. 10.21037/atm.2020.02.102 32355796 PMC7186700

[17] Lu X Jin J Lin J Qian W Weng X. Course of fever and potential infection after total joint replacement. Knee Surg Sports Traumatol Arthrosc. (2015) 23:1870–6. 10.1007/s00167-014-3098-y 24902928

[18] Kennedy JG Rodgers WB Zurakowski D Sullivan R Griffin D Beardsley W. Pyrexia after total knee replacement. A cause for concern? Am J Orthop. (1997) 26:549–52. 9267555

[19] Ishii Y Noguchi H Takeda M Sato J Takayama S Toyabe S. Characteristics and significance of fever during 4 weeks after primary total knee arthroplasty. Arch Orthop Trauma Surg. (2014) 134:707–12. 10.1007/s00402-014-1949-0 24522863 PMC3990857

[20] Pan JK Hong KH Xie H Luo MH Guo D Liu J. The efficacy and safety of autologous blood transfusion drainage in patients undergoing total knee arthroplasty: a meta-analysis of 16 randomized controlled trials. BMC Musculoskelet Disord. (2016) 17:452. 10.1186/s12891-016-1301-7 27806693 PMC5094026

[21] Andres BM Taub DD Gurkan I Wenz JF. Postoperative fever after total knee arthroplasty: the role of cytokines. Clin Orthop Relat Res. (2003) 221–31. 10.1097/01.blo.0000093914.26658.55 14612649

